# Effectiveness of virtual reality compared to video training on acetabular cup and femoral stem implantation accuracy in total hip arthroplasty among medical students: a randomised controlled trial

**DOI:** 10.1007/s00264-023-06038-8

**Published:** 2023-11-23

**Authors:** Eustathios Kenanidis, Panagiotis Boutos, Grigorios Voulgaris, Aikaterini Zgouridou, Eleni Gkoura, Zakareya Gamie, George Papagiannakis, Eleftherios Tsiridis

**Affiliations:** 1 Academic Orthopaedic Department, Aristotle University Medical School, General Hospital Papageorgiou, Ring Road Efkarpia, 56403 Thessaloniki, Greece; 2 Centre of Orthopaedic and Regenerative Medicine (CORE), Center for Interdisciplinary Research and Innovation (CIRI)-Aristotle University of Thessaloniki (AUTH), Balkan Center, Buildings A & B, Thessaloniki, 10th km Thessaloniki-Thermi Rd, P.O. Box 8318, 57001 Thessaloniki, GR Greece; 3 Institute of Computer Science, Foundation for Research and Technology (FORTH), Heraklion, Greece; 4 Department of Computer Science, University of Crete, Heraklion, Greece

**Keywords:** Virtual reality, VR, Hip arthroplasty, THA, Training, Randomized controlled trial

## Abstract

**Purpose:**

Virtual reality (VR) training effectiveness in improving hip arthroplasty surgical skills requires further evaluation. We hypothesised VR training could improve accuracy and the time taken by medical students compared to a control group with only video teaching.

**Methods:**

This single-centre randomized controlled clinical trial collected data from March to June 2023. Surgically naïve volunteer undergraduate medical students performed three sessions on a VR training platform, either cup (VR-Cup=Control-Stem) or stem (VR-Stem=Control-Cup) implantation. The primary outcome was the mean difference between predefined cup inclination (60°) and stem anteversion (20°) compared to the actual implanted values in sawbones between VR and control groups. Secondary outcomes were task completion time and mistake number between the groups.

**Results:**

A total of 101 students participated (VR-Cup 47, VR-Stem 54). Groups did not significantly differ concerning age (*p* = 0.879), gender (*p* = 0.408), study year (*p* = 0.938), previous VR use (*p* = 0.269) and baseline medical and procedural knowledge. The VR-Cup implanted the cup closer to the intended target (*p* < 0.001) and faster than the Control-Cup group (*p* = 0.113). The VR-Stem implanted the stem closer to the intended target (*p* = 0.008) but not faster than the Control-Cup group (*p* = 0.661). Stem retroversion was commoner in the Control-Stem than in the VR-Stem group (*p* = 0.016).

**Conclusions:**

VR training resulted in higher rates of accurate procedure completion, reduced time and fewer errors compared to video teaching. VR training is an effective method for improving skill acquisition in THA.

**Trial registration:**

ClinicalTrials.gov Identifier: NCT05807828

**Supplementary Information:**

The online version contains supplementary material available at 10.1007/s00264-023-06038-8.

## Introduction

Training medical students and junior surgeons is currently a priority in the medical field [1]. It is widely accepted that becoming proficient in surgery requires practical experience in the operating room and a deep understanding of the theoretical aspects [2]. The skill set requires time to develop through consistent practice, helping to develop hand-eye coordination [1, 3]. The orthopaedic techniques’ continuous evolution and the vast array have made this process more challenging [2, 4, 5].

All residents traditionally acquire new surgical skills by studying technique guides, watching procedure videos and working with a mentor [2, 6]. However, up to 96% of residents learn how to prepare for surgical procedures independently [7], and less than 80% of general surgery residents who have completed their training were considered fully competent [8]. Simulation use (sawbones’ use, cadaveric dissection, wet lab simulation models and navigation-based training) can support learning curves [9–11]. Limitations include single-use, availability and cost [12–14]. Virtual reality (VR) technology has been evaluated as a training method with promising results [15, 16]. However, there are a limited number of adequately powered studies on how it can be used to train surgeons in total hip arthroplasty (THA), with most studies relating to arthroscopy skills or trauma [15, 16]. It is unclear whether surgeons using VR gain advantages in identifying anatomical structures, understanding the steps involved in a procedure, improving their skills during difficult stages of a task, or more efficiently navigating themselves during surgery.

We designed a prospective randomised controlled trial (RCT) to measure if VR simulation helps medical students with no prior surgical experience acquire surgical technical skills and competence in THA by performing two core steps: the acetabular cup and femoral stem implantation in a specific position. This study hypothesised that VR training could help young doctors orient themselves in space and perform more accurate cup implantation at 60° inclination and femoral stem at 20° anteversion compared to a control group with only video teaching. The primary outcome was (a) the difference in the mean implanted cup inclination and femoral stem anteversion between the VR and control group and (b) the mean difference between the asked predefined and the actual implanted cup inclination or cup version between the VR and control groups. Secondary outcomes were the differences in completing the task between groups: (a) the time needed to complete the task and (b) the mistakes’ number.

## Materials and methods

### Recruitment

Our Institutional Review Board approved a prospective RCT (70/2023- 15/02/2023) undertaken from 26/03/23 and completed on 30/06/23. The study was registered in the Clinical Trials under the registration number NCT05807828. Eligible study participants included undergraduate medical students at our university with no previous surgical experience willing to participate. Exclusion criteria included (a) postgraduate medical students, (b) prior experience in THA or general surgery and (c) undergraduate students unwilling to participate. One month before the planned VR-THA surgery session, all eligible medical students were invited to an information session regarding the study through the university’s social media. The medical students who fulfilled the inclusion criteria were invited to participate following informed consent. We used the CONSORT checklist when writing our report [17].

### Pretest

Immediately after enrollment, medical students were asked to complete a multiple-choice pretest to assess baseline VR experience and hip arthritis and THA knowledge. It focussed on hip joint basic anatomical knowledge, hip arthritis and the THA basic steps and implant types. All participants were unaware and unable to prepare for the pretest assessment, which allowed the test scores to be considered an accurate measure of baseline knowledge (Appendix).

### Video teaching

All medical students were then asked to watch a detailed video explaining the hip joint basic anatomy, the hip arthritis fundamental pathophysiology and the THA steps. All participants were free to watch the video two to three times to understand the cup and femoral stem implantation principles thoroughly.

### Randomisation

**Fig. 1:**
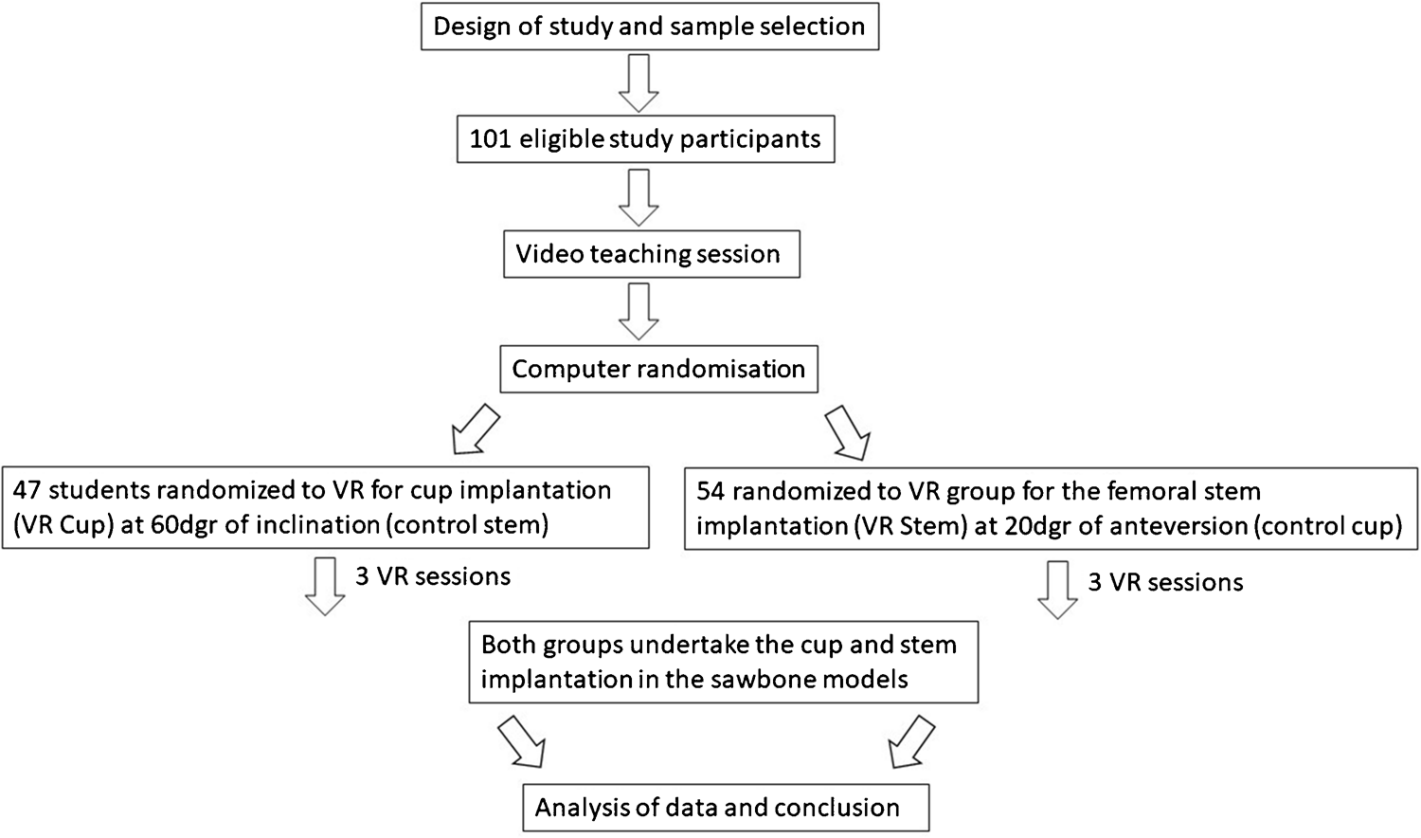
Study workflow of group randomisation and order of tasks undertaken

A computerised random number generator randomly assigned participants to the VR or control group for cup and stem training and implantation. In detail, each participant was randomised in the VR group for the cup or the stem implantation but in the control group for the other THA part. Therefore, participants enrolled on the VR group for cup (VR-Cup) training were the control group for the femoral stem (Control-Stem) training and vice versa. In this way, each participant was asked to do one implantation following VR training and the other without VR training. Participants were privately notified of their randomisation assignment and asked not to disclose their designated cohort with any other study participant or research personnel. Only a research team member knew of the cohort assignments during this study (P.B.). The study personnel did not participate in the evaluation and data analysis. The study workflow is summarized in Fig. 1.

### VR training

**Fig. 2:**
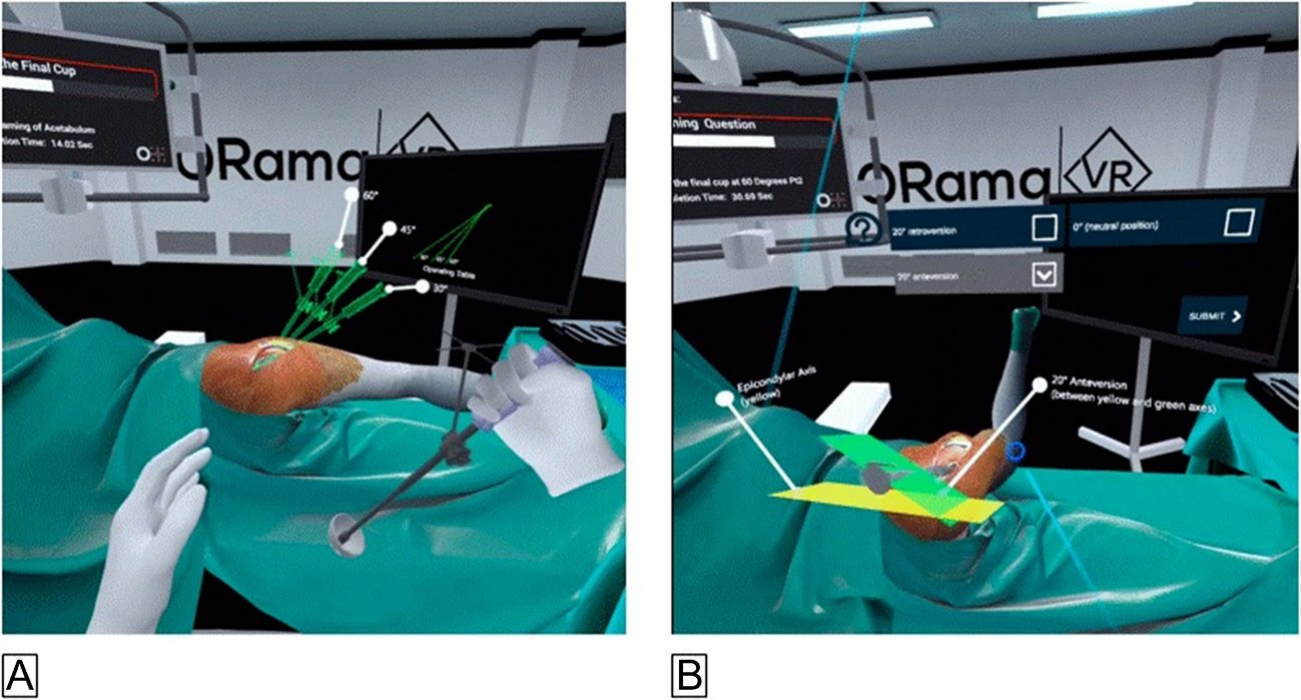
Virtual reality simulated operating room. **A** Acetabular cup implantation with immediate feedback of the inclination angle. **B** Femoral stem implantation with a marker for the epicondylar axis and immediate feedback of stem version

Each participant was randomised and performed one implantation (cup or stem) following VR training and the other (stem or cup) without VR training. This method allowed us to compare the performance of participants who received VR training versus those who did not for the same implantation scenario and the individual participants’ performance in both scenarios (stem or cup), with and without VR training. Before the VR-THA training, participants were asked to complete a survey evaluating their previous video game and VR technology experience. All VR group participants were then asked to complete three consecutive VR sessions using the VR system (ORama VR, Geneva, Switzerland), performing cup or stem implantation based on their group. This VR THA platform was used previously in another cadaveric study (Figs. 2A and 2B) [18].

### Saw bone simulation, cup inclination and femoral stem version measurement

After completing VR training, all participants were asked to implant the cup at a 60° inclination in sawbones and the femoral stem at 20° of anteversion. The acetabulum and the femur had been previously reamed and broached, respectively, by a senior orthopaedic surgeon to accommodate the metal prostheses from the students steadily. We used the appropriate size broach in the pre-broached saw bone and asked the students only to implant the broach at a special angle, and we measured that angle. The cup inclination and stem version were evaluated using goniometers and performed by study personnel unaware of the participant's group assignment. The time needed for implantation from each participant was recorded.

**Fig. 3:**
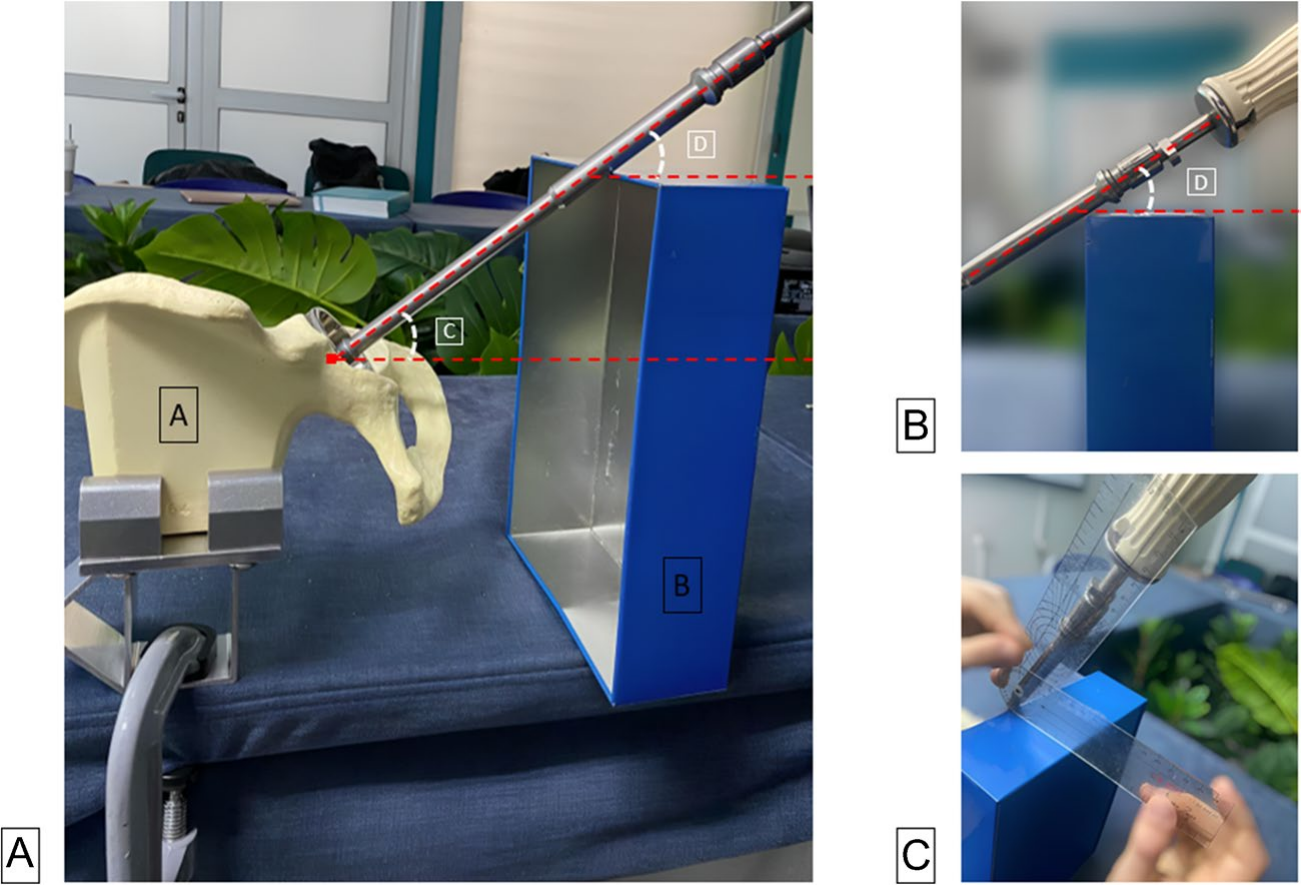
Cup implantation measurement. **A** Set-up of the hemipelvis (A) and the insertion handle and angles (C) and (D) formed in relation to the hemipelvis and quadrilateral box (B), respectively. **B** The angle (D) formed between the longitudinal axis of the insertion handle and the upper surface of the box. **C** Use of the goniometer to measure the angle formed

To determine the cup inclination angle, we utilised a hemipelvis sawbone, a clamp, an acetabular cup with an insertion handle and a standard goniometer (Fig. 3A). A clamping device securely held the sawbone on the table, mimicking the human pelvis placement in a lateral decubitus position during THA. After the student inserted the cup, a quadrilateral box was placed on the table near the insertion handle. The angle of the cup inclination was measured relative to the ground. The cup insertion angle (angle A) refers to the angle formed between the insertion handle’s longitudinal axis and the ground, which is parallel to the table and the upper box surface (angle B) (Fig. 3B). Angle A is like angle B due to parallelism. To facilitate measurement, angle B was used (Fig. 3C).

**Fig. 4:**
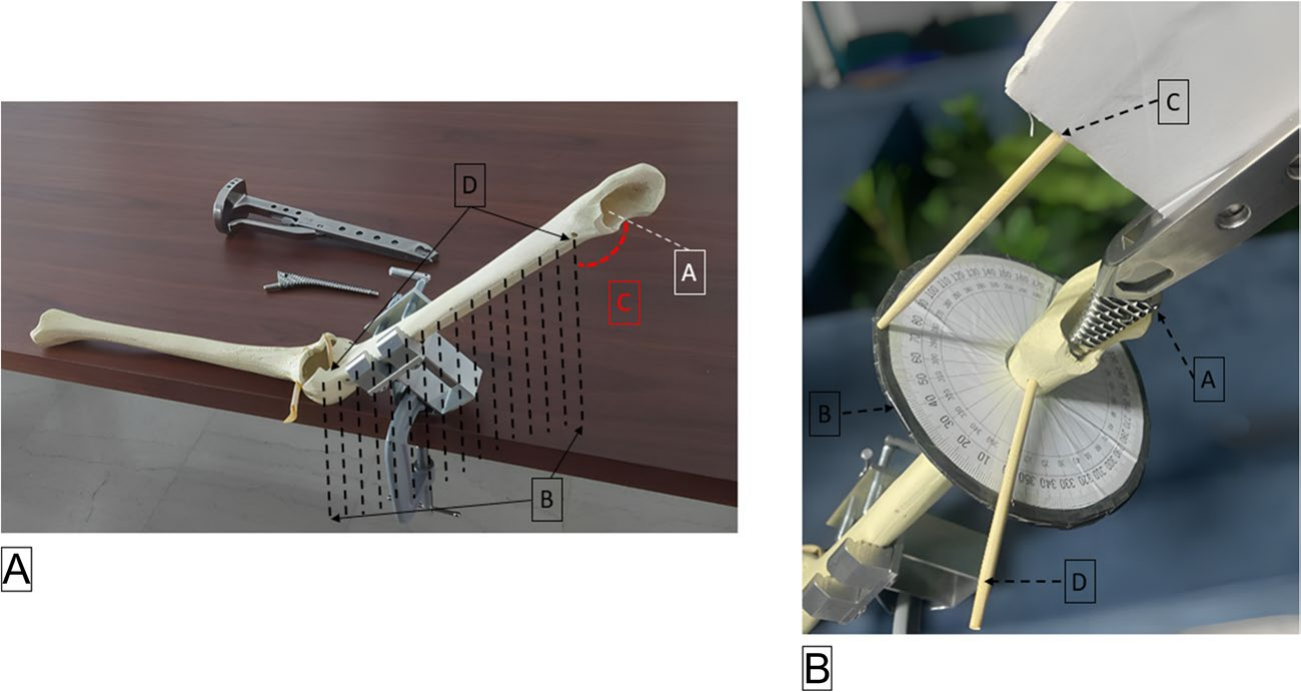
Femoral stem implantation measurement. **A** (A) The line representing the femoral neck plane. (B) The lines representing the plane passing through the middle of the femur and the two epicondyles. (C) The angle of the femoral stem version. (D) The two drill holes demarcating the transcondylar femoral plane. **B** (A) The insertion handle attached to the femur. (B) The ring goniometer. (C) The stick indicator designating the transepicondylar axis plane in the middle of the femur. (D) A piece of cardboard extension with a stick attached was placed in the middle of the insertion handle. This stem was inserted in 70° of retroversion

To determine the femoral stem version angle, we used a femoral sawbone, a tool to secure the sawbone in place on the table and a femoral rasp with an insertion handle. The femoral sawbone was positioned on the device to imitate the leg positioning during femoral canal preparation in ΤΗΑ performed through a posterior approach (Fig. 4A). The femoral version refers to the rotation angle between the femoral neck stem and the knee transcondylar axis. Once the student had inserted the femoral stem, a device was constructed to measure the angle from the transepicondylar axis plane, placed on 0° on the ring goniometer, and the insertion handle’s rod pointed to the femoral stem version angle (Fig. 4B).

### Power analysis

After reviewing eight recent RCT studies on VR simulation training [18–25], it was found that each study had an average of 14 to 28 participants and an effect size of 0.80 to 0.90. Previous studies have utilised specific scores to evaluate the trauma impact or arthroplasty simulator training on actual performance in real-world scenarios. These evaluations were different from our measurements. To assess our study’s necessary sample size, we based on the first ten cup angle implantation measurements from our medical students; the mean cup angle was 50.4° with a standard deviation of 18°. To detect a difference of 20° of implantation between two groups with a standard deviation of 18° and with a two-sided alpha of 0.05 and 80% power, at least 26 students were required in its group.

### Statistical analysis

The primary outcome was the mean implanted cup inclination and femoral stem anteversion difference between the VR and control groups. Other outcomes considered between groups included a) the duration required to complete the task and b) the number of errors made.

We used standard statistical methods to gather descriptive statistics. We conducted the Kolmogorov-Smirnov and Shapiro-Wilk tests to check whether the data followed a normal distribution. Our statistical tests were two-tailed, and we set the alpha level at 0.05. For continuous variables that were not normally distributed, we used the Mann-Whitney *U*-test. We compared categorical data using the chi-squared test. A paired *t*-test was conducted to compare their outcomes with and without VR to determine if using VR impacted a student’s task performance. We performed all statistical analyses using SPSS software (IBM, version 27.0).

## Results

### Demographic comparative data

One hundred and one undergraduate medical students were enrolled and completed the study. Fifty-six men and 45 women with a mean age of 23.3 (2.9) years participated in the study. The mean age, sex prevalence, study year, previous VR technology experience and baseline medical and procedural knowledge of hip arthritis and THA did not differ significantly between groups (Table 1).

**Table 1:** Comparative demographic and questionnaire data between groups.

		VR-Cup (control stem group)	VR-Stem (control cup group)	*p*
Sex^*^	Men	24 (51.0) 23 (48.9)	32 (59.2) 22 (40.7)	0.408^#^
	Women			
Age^**^ (years)		23.49 (3.0)	23.26 (2.8)	0.879^@^
Academic study year^*^	1^st^	3 (6.3) 4 (8.5) 4 (8.5) 13 (27.6) 19 (40.4) 4 (8.5)	2 (3.7) 5 (9.2) 7 (12.9) 12 (22.2) 24 (44.4) 4 (7.4)	0.938^#^
	2^nd^			
	3^rd^			
	4^th^			
	5^th^			
	6^th^			
VR questionnaire				
Prior use of VR in any training program^*^	Yes	3 (6.4) 44 (93.6)	7 (13) 47 (87)	0.269^#^
	No			
Prior use of VR glasses for any reason^*^	Yes	16 (34.0) 31 (66.0)	28 (52) 26 (48)	0.072^#^
	No			
Ownership of VR headset^*^	Yes	0 (0) 47 (100)	2 (3.8) 52 (96.2)	0.183^#^
	No			
Knowledge of VR technology prior to this study^*^	Yes	47 (100) 0 (0)	53 (98.1) 1 (1.2)	0.348^#^
	No			
Knowledge and previous training questionnaire				
Basic arthroplasty question concerning the typical parts of THA	Correct answer	37 10	45 9	0.554^#^
	Wrong answer			
Basic pathophysiology OA question concerning the typical damage during OA	Correct answer	38 9	42 12	0.704^#^
	Wrong answer			
Previous attendance of an orthopaedic surgery	Yes	10 37	16 38	0.338^#^
	No			
Previous exposure to any surgical procedure (other than orthopaedic)	Yes	10 37	15 39	0.450^#^
	No			
Basic arthroplasty question concerning fixation methods in THA	Correct answer	11 36	11 43	0.713^#^
	Wrong answer			
Basic question concerning the typical cup implantation angle during THA	Correct answer	8 39	5 49	0.245^#^
	Wrong answer			
Basic question concerning the typical stem version implantation during THA	Correct answer	0 47	1 53	0.348^#^
	Wrong answer			

*VR* virtual reality, *THA* total hip arthroplasty, *OA* osteoarthritis

^*^The values are given as raw numbers with percentages in parentheses

^**^The values are given as the mean with the standard deviation (±) in parentheses

^@^Test was performed using the Mann-Whitney test

^#^Test was performed using *x*^2^ test

### Cup implantation data

The median (interquartile range, IQR) cup inclination of the VR-Cup group 60° (9°) was significantly closer to the intended surgical target than the Control-Cup group 52.5° (12.4°) (Mann-Whitney test, *p* < 0.001). The difference between the predefined and the implanted cup inclination was also significantly different, favouring the VR-Cup (4° (7°) vs. 8° (12°)) (median, (IQR), Mann-Whitney test, *p* < 0.001). The time taken for the VR-Cup group was shorter, 21 (25) sec compared to the control, 35 (27) sec, but this difference was not statistically significant (median (IQR), Mann-Whitney test, *p* = 0.113).

### Femoral stem implantation data

The median (interquartile range, IQR) femoral stem version in the VR-Stem group 27.5° (276°) was significantly closer to the intended surgical target than the Control-Stem group 319° (317°) (Mann-Whitney test, *p* = 0.008). The difference between the predefined and the implanted femoral stem version was also significantly different, favouring the VR-Stem (17° (263°) vs. 275° (305°)) (median (IQR), Mann-Whitney test, *p* = 0.008). The medical students’ number that put the stem in retroversion than anteversion was significantly greater in the Control-Stem than in the VR-Stem group (17 (31.4%) vs. 26 (55.31%), *x*^2^ test, *p* = 0.016). The VR-Stem group required an equal time of 43 (46) sec than the Control-Stem group of 45 (37) sec (*p* = 0.661).

### Comparative data for each medical student with or without VR

Each medical student carried out an implantation following VR training and without VR training. Therefore, there was a deviation between the predefined target and the implanted inclination for the cup and the stem version for the same medical student. Comparing these differences for the same student, we found that the mean difference for students using VR before implantation was 54.5° ± 106.6° and without VR training, 89.8° ± 133.9°. This difference was statistically different, favouring the VR group (paired *t*-test, *p* = 0.004).

## Discussion

The study aimed to investigate if VR could improve cup and femoral stem implantation precision by inexperienced medical students. They involve a range of skills such as planning, human anatomy knowledge and visuospatial orientation that are crucial in orthopaedic reconstructive surgery [2, 18]. VR resulted in higher rates of accurate procedure completion, reduced time and fewer errors compared to relying solely on a video teaching method.

VR studies on critical THA elements influencing outcomes, such as cup and femoral stem implantation accuracy, are limited. Studies by Logishetty et al. have reported on VR use for THA and found improved acetabular component positioning, femoral osteotomy angle, procedure duration and global competency for the key steps in the VR group [5]. Secondary outcomes also favour VR, including error rates and procedure duration. A subsequent THA study found faster task completion in the VR group [5], and pooled data in a meta-analysis found no notable differences in global competence with VR; however, the procedure duration was shorter [10]. Most studies investigating VR have measured procedure duration, used measurement tools for global competency assessment and may not include key elements of surgical competencies that influence the outcome [1, 3]. Unique to our study was assessing the femoral component version, which has been less studied; we found significantly higher accuracy with VR use and that the time for task completion was longer than acetabular component placement as it was found to be more challenging.

A systematic review of head-mounted display VR in a range of surgical procedures such as shoulder arthroplasty and glenoid exposure (19 residents/seven consultant surgeons), reverse shoulder arthroplasty (18 residents), pedicle screw placement (24 surgeons) and tibial intramedullary nailing (25 students) has found VR to be an effective tool at improving learning efficiency, knowledge, skill transfer and at reducing errors made and improving skills [9]. More recently, a meta-analysis including four RCTs and one prospective controlled study looked explicitly at extended reality use for THA ranging from 7 to 32 participants (106 in total, mainly surgeons) utilising different hip approaches in models such as a cadaver and dry bone model, an augmented reality (AR) phantom hip model and a radiopaque foam pelvis [11]. The studies investigated acetabular cup position inclination and version. The review found the average inclination value ranged from 1.8 to 4° and control 4.8 to 15° (mean 4.89° VR vs. 10.91° control in our study), and surgical duration was lower in the VR group in two of the three studies that reported this [11]. After removing one study after sensitivity analysis due to significant heterogeneity, the pooled data found higher accuracy for inclination in the VR group. We also found mean cup implantation time quicker with VR at 31 sec. The pooled data for anteversion, which was significantly heterogeneous, found similar accuracy with VR vs. conventional. Further study is required here and demonstrates the challenges with assessing the version measurement accuracy. Overall, there were five studies with 106 participants, including a non-randomised study that weakened the study’s findings. Furthermore, no significant effect on medical knowledge was found compared to technical skills. Our study has, therefore, approximately doubled the trial evidence to date for the VR use for the component alignment accuracy.

### Practical implications and limitations

VR technology has immense potential to benefit surgical residency programs [26] and various VR limitations. This technology’s tactile, haptic feedback is currently better suited for laparoscopic procedures and needs improvement to simulate larger open orthopaedic procedures effectively. It is necessary to thoroughly evaluate the simulators’ cost to their long-term potential advantages. They have been considered cost-effective in other disciplines and surgical procedures; a recent paper investigating a VR platform for reverse shoulder arthroplasty concluded that it is cost-effective from the reduction in surgical training time, which can reduce costs associated with complications [25].

Research is necessary to determine the optimal VR use in teaching orthopaedic procedures, as there are many orthopaedic procedures. Which procedures should be prioritized for simulation using VR? Furthermore, the amount of work required from simulation platforms to code this needs to be considered.

We recognise this study’s limitations, such as the medical students’ recruitment and not junior resident surgeons, which may be considered more appropriate for this study due to the level of competence. However, residents may have other variables and substantial confounders as they learn from other sources (videos and technique guides) and are variably exposed to surgery. Medical students are entirely naïve to these procedures, easily recruited with no prior exposure to surgical operations and are, therefore, a more homogenous group and do not undermine the study’s validity and applicability. Sawbone models and the VR environment do not accurately represent a surgical scenario. The surgical environment presents several haptic, technical and situational awareness challenges that are not easily replicated.

VR can potentially be a valuable tool in surgical residencies for learning procedures, and practising required surgical movements and visuospatial orientation. By providing an alternative training method, VR can serve as a helpful supplement to traditional training methods. Surgical residents have encountered increasing challenges and time taken to train and work independently. Using a training tool to reduce errors during practice can improve their skills and ultimately benefit patient safety.

### Supplementary information

ESM 1 (DOCX 19 kb)

ESM 2 (DOCX 26 kb)
