# Supplementary material for: Effectiveness of virtual reality compared to video training on acetabular cup and femoral stem implantation accuracy in total hip arthroplasty among medical students: a randomised controlled trial
Source: Int Orthop. 2023 Nov 23;48(3):625–33. doi: 10.1007/s00264-023-06038-8 (PMC10901922; doi:10.1007/s00264-023-06038-8)
Supplement: Supplementary file 1 — (DOCX 19 kb) [file 264_2023_6038_MOESM1_ESM.docx]

QUESTIONNAIRE

GENERAL:

- Study Year: _________

- Age: _________
- Sex:
  1. Male
  2. Female
  3. Other

VIRTUAL REALITY:

- Have you ever taken part in a training program that utilizes virtual reality (VR) technology?
  1. YES
  2. NO
- Have you ever tried wearing virtual reality (VR) glasses?
  1. YES
  2. NO
- Do you happen to possess a virtual reality headset?
  1. YES
  2. NO
- Did you know about VR technology prior to learning about this research?
  1. YES
  2. NO

HIP ARTHROPLASTY:

- A typical total hip arthroplasty includes:

1. Femoral stem
2. Acetabular cup
3. Femoral head
4. Polyethylene
5. All of the above
6. None of the above

- The typical damage of joint hip osteoarthritis involves:
  1. Articular cartilage
  2. Synovial membrane
  3. Synovial fluid
  4. Joint ligaments
  5. None of the above
- Have you ever attended/undergone an orthopaedic surgery?
  1. YES (Type of surgery: ……………………………………………………….)
  2. NO
- Have you been trained in any surgical procedures (other than orthopaedic)?
  1. YES (Type of surgery: ……………………………………………………….)
  2. NO
- During total hip arthroplasty, the implants can be fixed in place using...:
  1. Biological cement
  2. Osteosynthesis with plate and screws
  3. External osteosynthesis
  4. All of the above
  5. None of the above
- The typical inclination of the acetabular cup in total hip arthroplasty is (degrees): …………………………………………………………..
- The typical version of the femoral stem in total hip arthroplasty is (degrees)…………………………………………………………………………………….

Appendix 1. Questionnaire to assess baseline Virtual Reality (VR) experience and knowledge of hip arthritis and arthroplasty.
